# Supplementary figures and images for: An atomic interaction conserved for over 600 million years gates inhibitory neurotransmission
Source: bioRxiv. 2026 May 26:2026.05.22.727206. Preprint. [Version 1] doi: 10.64898/2026.05.22.727206 (PMC13232294; doi:10.64898/2026.05.22.727206)

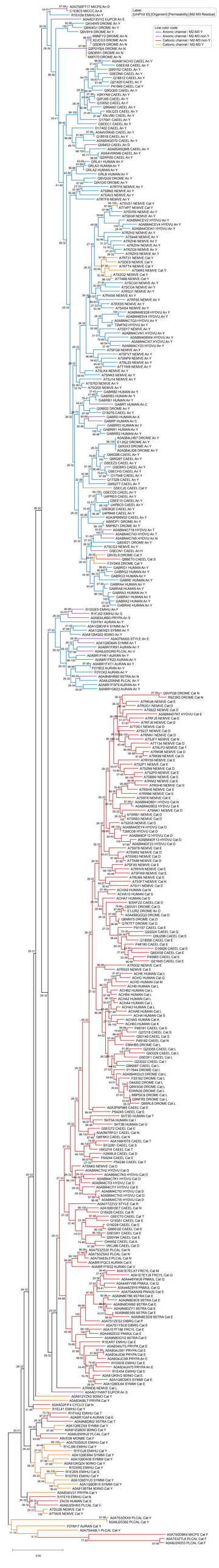

Supplement: Supplement 1 [file media-1.pdf]
