# Supplementary material for: An atomic interaction conserved for over 600 million years gates inhibitory neurotransmission
Source: bioRxiv. 2026 May 26:2026.05.22.727206. Preprint. [Version 1] doi: 10.64898/2026.05.22.727206 (PMC13232294; doi:10.64898/2026.05.22.727206)
Supplement: Supplement 2 [file NIHPP2026.05.22.727206v1-supplement-2.pdf]

bioRxiv preprint doi: https://doi.org/10.64898/2026.05.22.727206; this version posted May 26, 2026. The copyright holder for this preprint (which was not certified by peer review) is the author/funder, who has granted bioRxiv a license to display the preprint in perpetuity. It is made available under a CC-BY 4.0 International license.

Supplementary File 1

Phylogenetic tree of pLGICs, identifiable as forming either anionic or cationic channels, from unicellular and multicellular eukaryotes. The tree was constructed using the maximum likelihood method, with the number of replicates (196) determined adaptively. The percentage of replicate trees in which the associated taxa clustered together is shown next to the branches. The bootstrap range values represent the last estimated bootstrap support value for that branch - the upper bound (or confidence limit) estimated during the adaptive bootstrap procedure. The branch labels indicate [UniProt ID] [organism code] [anion-/cation-selectivity] [M2-M3 residue at the homologous position with the conserved tyrosine]. The organism name codes can be found in Supplementary Table 4. The color of the lines indicates anion-/cation-selectivity and the M2-M3 residue (blue: Anionic/Tyrosine; violet: Anionic/Non-tyrosine; red: Cationic/Non-tyrosine; orange: Cationic/Tyrosine).

bioRxiv preprint doi: https://doi.org/10.64898/2026.05.22.727206; this version posted May 26, 2026. The copyright holder for this preprint (which was not certified by peer review) is the author/funder, who has granted bioRxiv a license to display the preprint in perpetuity. It is made available under a CC-BY 4.0 International license.

Supplementary Information for "An atomic interaction conserved for over 600 million years gates inhibitory neurotransmission"

Cecilia M. Borghese[1], Netrang G. Desai[1], Pranavi Garlapati[1], Syedah K. Shah[1], Jason D. Galpin[2], Luthary Segura[1], Nandan Haloi[3], Harold H. Zakon[1,4], Rebecca J. Howard[3,5], Erik Lindahl[3,5-7], Christopher A. Ahern[2], and Marcel P. Goldschen-Ohm[1]*

[1]Department of Neuroscience, The University of Texas at Austin, Austin, TX, USA
[2]Department of Molecular Physiology and Biophysics, University of Iowa, Iowa City, IA, USA
[3]Department of Biochemistry and Biophysics, Stockholm University, Stockholm, Sweden
[4]Department of Integrative Biology, and Biodiversity Center, The University of Texas at Austin, Austin, TX, USA
[5]Department of Applied Physics, KTH Royal Institute of Technology, Stockholm, Sweden
[6]Department of Physics, Chemistry and Biology, Linköping University, Linköping, Sweden
[7]Department of Chemistry, University of Illinois at Urbana-Champaign, Urbana, IL, USA

Content

Four figures and four tables

bioRxiv preprint doi: https://doi.org/10.64898/2026.05.22.727206; this version posted May 26, 2026. The copyright holder for this preprint (which was not certified by peer review) is the author/funder, who has granted bioRxiv a license to display the preprint in perpetuity. It is made available under a CC-BY 4.0 International license.

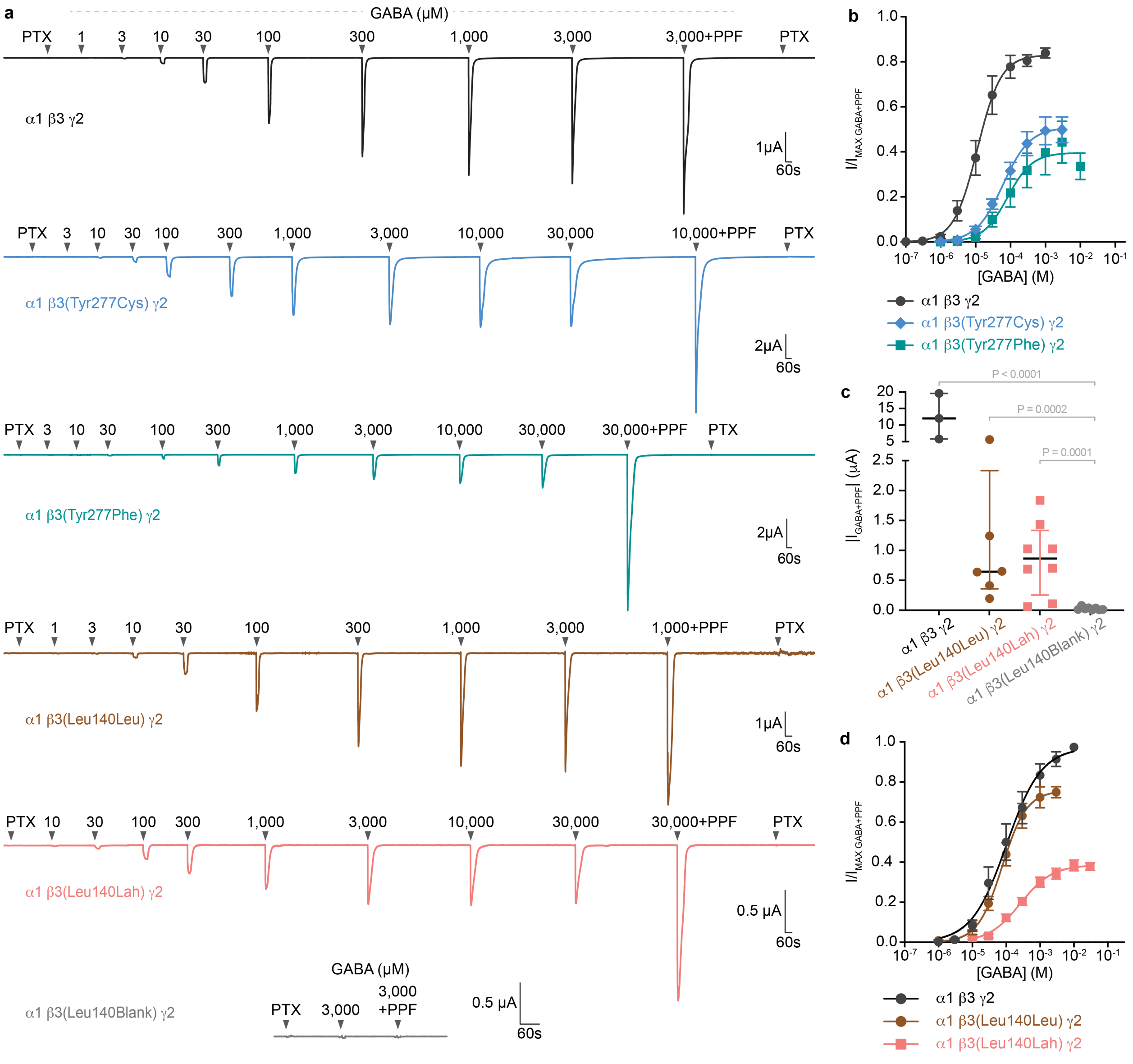

Supplementary Figure 1. A H-bond linking gating loops is functionally important in GABA$_A$ β subunits.

a Representative current traces for α1β3γ2 GABA$_A$Rs and either 1) substitutions of the conserved tyrosine β3(Tyr277) or 2) nonsense suppression incorporation of either wild-type leucine or its cognate α-hydroxy acid (Lah) at β3(Leu140). Arrows above traces indicate approximate onset of pulses of either 1 mM picrotoxin (PTX), increasing concentrations of GABA, or maximal GABA + 30 μM propofol (PPF). **b** Concentration-response relations for GABA-elicited currents, normalized to maximal GABA + PPF responses. Data are mean ± SEM across oocytes. Curves are fits of the Hill equation (Eq. 1) to the means. See Supplementary Table 3 for summary statistics and fit parameters. Number of oocytes (n) are: α1β3γ2; n = 6; α1β3(Tyr277Cys)γ2: n = 4; α1β3(Tyr277Phe)γ2: n = 6. **c** Total current per oocyte obtained in the presence of maximal GABA + 30 μM propofol suggests reliable nonsense suppression incorporation of Leu and Lah with little to no read-through (i.e., relative lack of current for Blank). Number of oocytes for each condition

bioRxiv preprint doi: https://doi.org/10.64898/2026.05.22.727206; this version posted May 26, 2026. The copyright holder for this preprint (which was not certified by peer review) is the author/funder, who has granted bioRxiv a license to display the preprint in perpetuity. It is made available under a CC-BY 4.0 International license.

(left to right) are n = 3, 6, 8, 8. Plots show median and interquartile intervals. P-values are from Brown-Forsythe ANOVA with posthoc Dunnett's T3 multiple comparisons test. **d** Concentration-response relations for GABA-elicited currents, normalized to maximal GABA + PPF responses. Data are mean ± SEM across oocytes. Curves are fits of the Hill equation (Eq. 1) to the means. See Supplementary Table 3 for summary statistics and fit parameters. Number of oocytes (n) are: α1β3γ2: n = 3; α1β3(Leu140Leu)γ2: n = 6; α1β3(Leu140Lah)γ2: n = 8.

bioRxiv preprint doi: https://doi.org/10.64898/2026.05.22.727206; this version posted May 26, 2026. The copyright holder for this preprint (which was not certified by peer review) is the author/funder, who has granted bioRxiv a license to display the preprint in perpetuity. It is made available under a CC-BY 4.0 International license.

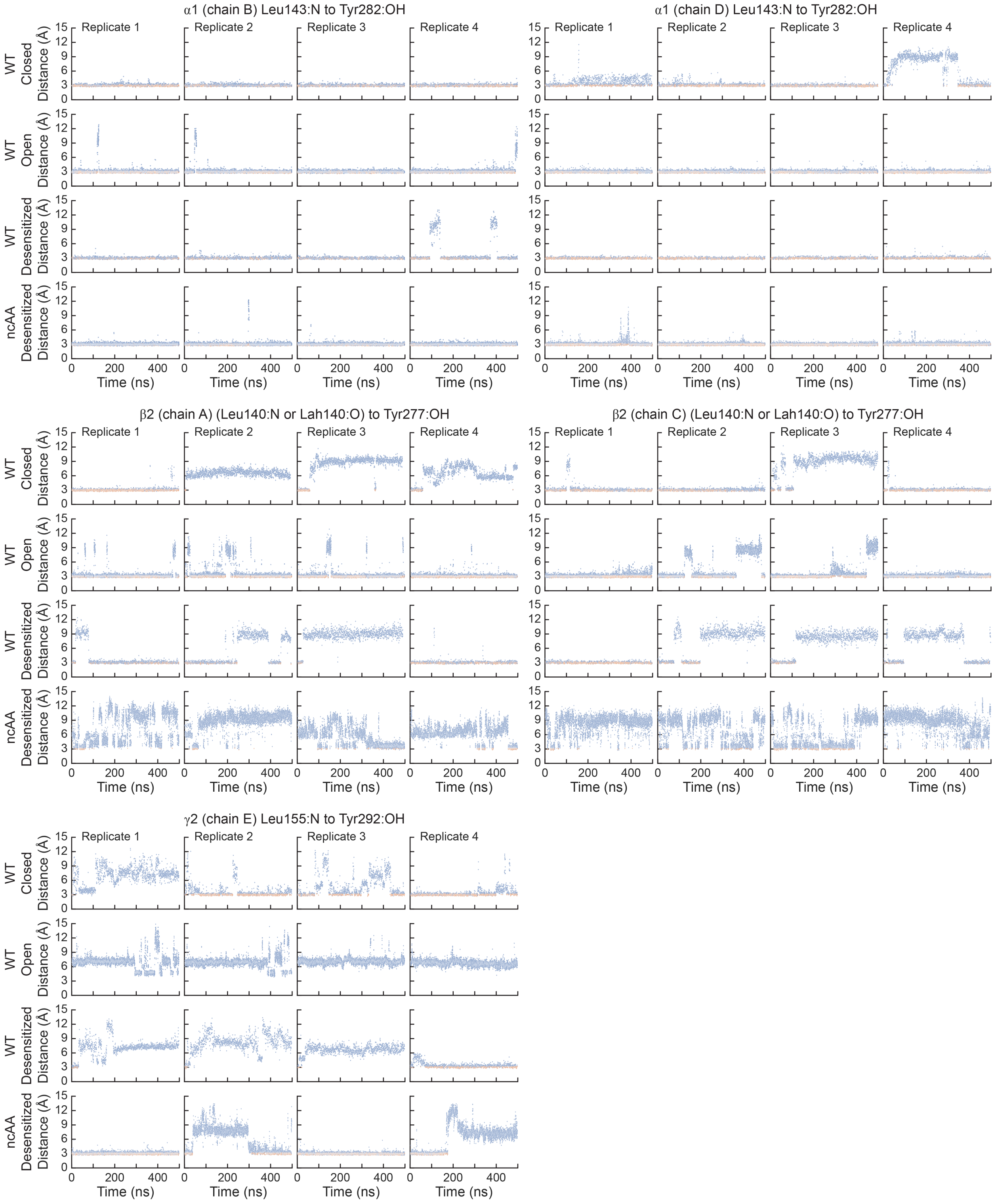

Supplementary Figure 2. Time-resolved H-bond linkage between gating loops.

Donor-acceptor distance trajectories from MD simulations of α1β2γ2 GABA$_A$Rs in closed (antagonist-bound, PDB 6X3S), open (simulated structure (Haloi et al. 2025)), and desensitized (GABA-bound, PDB 6X3Z) states with and without the β2(Leu140Lah) amide-to-ester ncAA

bioRxiv preprint doi: https://doi.org/10.64898/2026.05.22.727206; this version posted May 26, 2026. The copyright holder for this preprint (which was not certified by peer review) is the author/funder, who has granted bioRxiv a license to display the preprint in perpetuity. It is made available under a CC-BY 4.0 International license.

substitution. Distances are between a leucine backbone amide nitrogen in the Cys-loop (or the ester oxygen for the ncAA substitution) and a tyrosine OH group in the M2-M3 linker of the same subunit. Residue pairs are α1(Leu143:N-Tyr282:OH), β2(Leu140:N-Tyr277:OH), γ2(Leu155:N-Tyr292:OH), or β2(Lah140:O-Tyr277:OH). Data points are colored based on whether the atoms are within (red) or outside (blue) typical H-bond constraints (i.e., distance < 3.2 Å and bond angle in the range 120-150°).

bioRxiv preprint doi: https://doi.org/10.64898/2026.05.22.727206; this version posted May 26, 2026. The copyright holder for this preprint (which was not certified by peer review) is the author/funder, who has granted bioRxiv a license to display the preprint in perpetuity. It is made available under a CC-BY 4.0 International license.

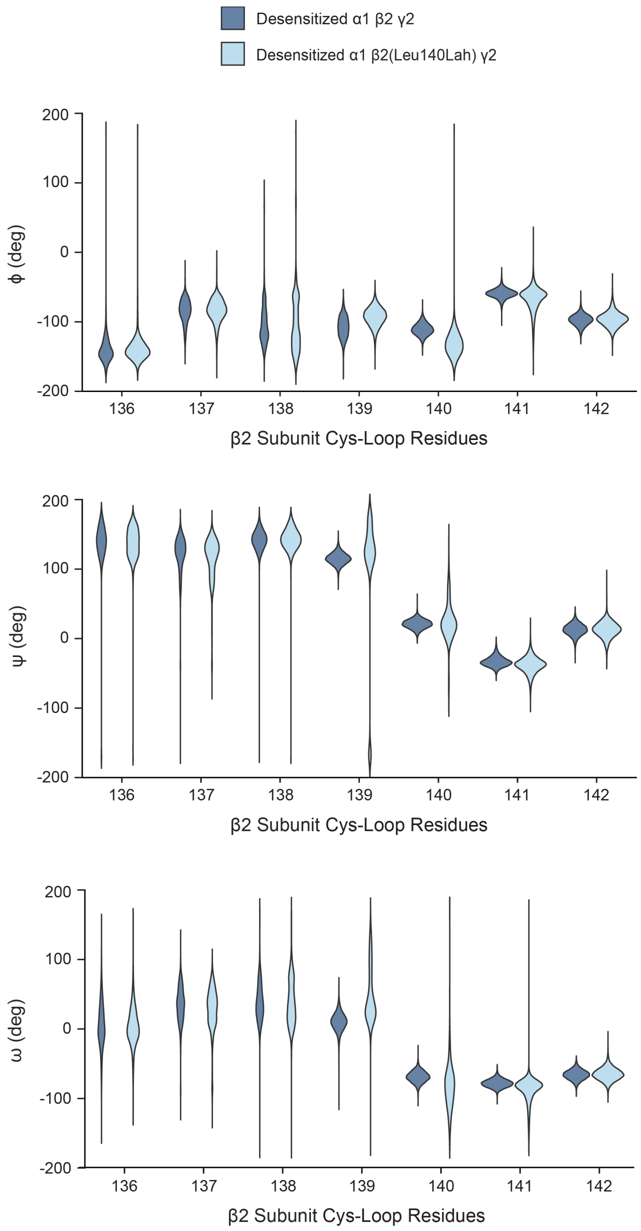

Supplementary Figure 3. Increased backbone flexibility for an amide-to-ester swap that ablates a stabilizing H-bond.

Backbone dihedral angle distributions ϕ, ψ, ω for Cys-loop residues 136-142 of β2 (chains A and C combined) from MD simulations of an α1β2γ2 $GABA_AR$ in a desensitized (i.e., GABA-bound, PDB 6X3Z) conformation with and without the β2(Leu140Lah) amide-to-ester ncAA substitution. ϕ is defined by the atoms $C_{i-1} - N_i - C_{\alpha i} - C_i$. ψ is defined by the atoms $N_i - C_{\alpha i} - C_i$ - N_{i+1}. ω is defined by the atoms $C_{\alpha i} - C_i - N_{i+1} - C_{\alpha i+1}$. For β2(Leu140Lah), the backbone nitrogen is replaced by the corresponding ester oxygen in these definitions.

bioRxiv preprint doi: https://doi.org/10.64898/2026.05.22.727206; this version posted May 26, 2026. The copyright holder for this preprint (which was not certified by peer review) is the author/funder, who has granted bioRxiv a license to display the preprint in perpetuity. It is made available under a CC-BY 4.0 International license.

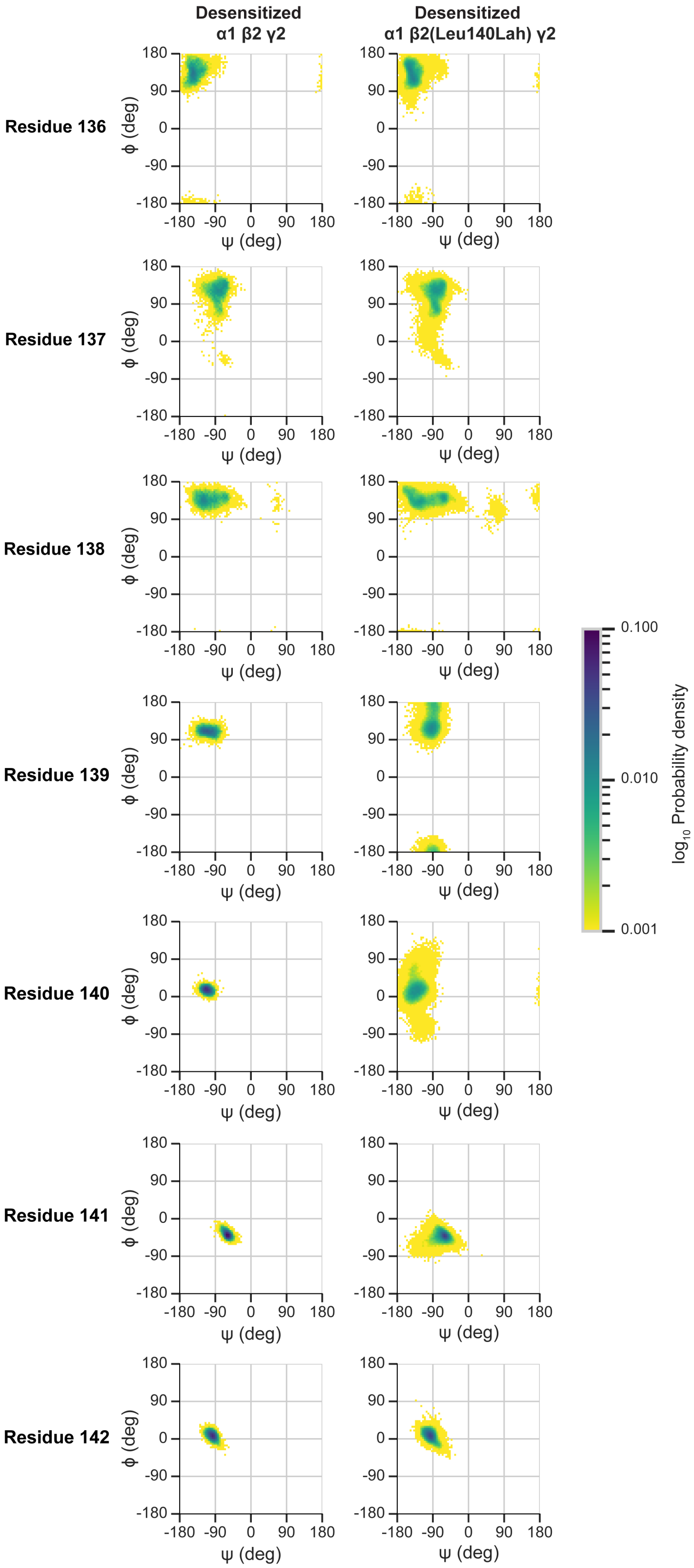

Supplementary Figure 4. Increased backbone flexibility for an amide-to-ester swap that ablates a stabilizing H-bond.

Ramachandran plots (Kleywegt and Jones 1996) of backbone dihedral angles ϕ and ψ for Cys-loop residues 136–142 of the β2 subunit (chains A and C combined) from MD simulations of an α1β2γ2 $GABA_AR$ in a desensitized (i.e., GABA-bound, PDB 6X3Z) conformation with and without the β2(Leu140Lah) amide-to-ester ncAA substitution. ϕ is defined by the atoms $C_{i-1} – N_i – C_{\alpha i} – C_i$. ψ is defined by the atoms $N_i – C_{\alpha i} – C_i$ - N_{i+1}. ω is defined by the atoms $C_{\alpha i} – C_i – N_{i+1} – C_{\alpha i+1}$. For β2(Leu140Lah), the backbone nitrogen is replaced by the corresponding ester oxygen in these definitions.

bioRxiv preprint doi: https://doi.org/10.64898/2026.05.22.727206; this version posted May 26, 2026. The copyright holder for this preprint (which was not certified by peer review) is the author/funder, who has granted bioRxiv a license to display the preprint in perpetuity. It is made available under a CC-BY 4.0 International license.

Supplementary Table 1. Sequence alignment of the Cys-loop and M2-M3 linker for inhibitory and excitatory pLGICs.

Protein	Organism	UniProt	Cys loop	M2-M3 linker
GABA$_A$ α1	Human	P14867	CPMH**L**EDFPMDAHAC	NSLPKVA**Y**-ATA
GABA$_A$ α2	Human	P47869	CPMH**L**EDFPMDAHSC	NSLPKVA**Y**-ATA
GABA$_A$ α3	Human	P34903	CPMH**L**EDFPMDVHAC	NSLPKVA**Y**-ATA
GABA$_A$ α4	Human	P48169	CPMR**L**VDFPMDGHAC	HSLPKVS**Y**-ATA
GABA$_A$ α5	Human	P31644	CPMQ**L**EDFPMDAHAC	NSLPKVA**Y**-ATA
GABA$_A$ α6	Human	Q16445	CPMR**L**VNFPMDGHAC	HSLPKVS**Y**-ATA
GABA$_A$ β1	Human	P18505	CMMD**L**RRYPLDEQNC	ETLPKIP**Y**-VKA
GABA$_A$ β2	Human	P47870	CMMD**L**RRYPLDEQNC	ETLPKIP**Y**-VKA
GABA$_A$ β3	Human	P28472	CMMD**L**RRYPLDEQNC	ETLPKIP**Y**-VKA
GABA$_A$ γ1	Human	Q8N1C3	CYLQ**L**HNFPMDEHSC	KSLPKVS**Y**-VTA
GABA$_A$ γ2	Human	P18507	CQLQ**L**HNFPMDEHSC	KSLPKVS**Y**-VTA
GABA$_A$ γ3	Human	Q99928	CQLQ**L**HNFPMDEHSC	KSLPRVS**Y**-VTA
GABA$_A$ δ	Human	O14764	CDMD**L**AKYPMDEQEC	SSLPRAS**A**-IKA
GABA$_A$ ε	Human	P78334	CSLH**M**LRFPMDSHSC	KNFPRVS**Y**-ITA
GABA$_A$ π	Human	O00591	CNMD**L**SKYPMDTQTC	TSLPNTN**C**FIKA
GABA$_A$ θ	Human	Q9UN88	CSLD**L**HKFPMDKQAC	DKLPNIS**C**-IKA
GABA$_A$ ρ1	Human	P24046	CNMD**F**SRFPLDTQTC	ASMPRVS**Y**-IKA
GABA$_A$ ρ2	Human	P28476	CNMD**F**SHFPLDSQTC	ASMPRVS**Y**-VKA
GABA$_A$ ρ3	Human	A8MPY1	CFMD**F**SRFPLDTQNC	ASMPQVS**Y**-LKA
GlyR α1	Human	P23415	CPMD**L**KNFPMDVQTC	ASLPKVS**Y**-VKA
GlyR α2	Human	P23416	CPMD**L**KNFPMDVQTC	ASLPKVS**Y**-VKA
GlyR α3	Human	O75311	CPMD**L**KNFPMDVQTC	ASLPKVS**Y**-VKA
GlyR β	Human	P48167	CPLD**L**TLFPMDTQRC	AELPKVS**Y**-VKA
RDL	*D. melanogaster*	P25123	CPMN**L**QYFPMDRQLC	AALPKIS**Y**-VKS
UNC-49	*C. elegans*	G5EBQ0	CPMD**L**KLFPMDSQHC	SSMPKVS**Y**-VKS
GluCL α	*C. elegans*	G5EBR3	CPMY**L**QYYPMDVQQC	SQLPPVS**Y**-IKA
GluCL β	*C. elegans*	Q17328	CPMR**L**QLYPLDYQSC	AKLPPVS**Y**-VKV
GLIC	*G. violaceus*	Q7NDN8	SPLD**F**RRYPFDSQTL	TNLPKTP**Y**-MTY
ZACN	Human	Q401N2	CNFE**L**LHFPRDHSNC	QALPSSSSCNPL
5-HT$_{3A}$	Human	P46098	CSLD**I**YNFPFDVQNC	DTLPATAIGTPL
nAChR α4	Human	P43681	CSID**V**TFFPFDQQNC	EIIPSTSLVIPL
nAChR α7	Human	P36544	CYID**V**RWFPFDVQHC	EIMPATSDSVPL
nAChR β1	Human	P11230	CSIQ**V**TYFPFDWQNC	DKVPETSLSVPI
nAChR α1	Human	P02708	CEII**V**THFPFDEQNC	ELIPSTSSAVPL
nAChR β2	Human	P17787	CKIE**V**KHFPFDQQNC	KIVPPTSLDVPL
nAChR β3	Human	Q05901	CTMD**V**TFFPFDRQNC	EIIPSSSKVIPL
nAChR β4	Human	P30926	CKIE**V**KYFPFDQQNC	KIVPPTSLDVPL
nAChR δ	Human	Q07001	CPIS**V**TYFPFDWQNC	KRLPATSMAIPL
nAChR ε	Human	Q04844	CAVE**V**TYFPFDWQNC	QKIPETSLSVPL
nAChR γ	Human	P07510	CSIS**V**TYFPFDWQNC	KKVPETSQAVPL

bioRxiv preprint doi: https://doi.org/10.64898/2026.05.22.727206; this version posted May 26, 2026. The copyright holder for this preprint (which was not certified by peer review) is the author/funder, who has granted bioRxiv a license to display the preprint in perpetuity. It is made available under a CC-BY 4.0 International license.

Supplementary Table 2. Variants in the M2-M3 position of inhibitory pLGICs subunits.

Receptor	Subunit	Substitution	Mutation	ClinVar	
$GABA_A$	α1	Y309>F	A>T	no report (COSV50102834)	Moderate Impact (predicted)
	α2	Y309>C	A>G	no report (NCI-TCGA: TCGA novel)	Probably damaging (predicted)
	α2	Y309>H	T>C	no report (COSV62915565)	no prediction
	α3	Y334>C	A>G	no report (NCI-TCGA: TCGA novel)	Moderate Impact (predicted)
	α4	Y315>D	T>G	no report (rs1184211379)	Probably damaging (predicted)
	α5	Y316>D	T>G	no report (COSV107422957)	no prediction
	α5	Y316>F	A>T	no report (COSV59476238)	no prediction
	α5	Y316>N	T>A	no report (rs1186567951)	Probably damaging (predicted)
	α6	Y299 none reported			
	β1	Y302 none reported			
	β2	Y301>C	A>G	RCV000479025, RCV000688521	Pathogenic
	β2	Y301>F	A>T	RCV001390027, RCV004798911	Pathogenic
	β2	Y301>H	T>C	RCV002035695	Pathogenic
	β3	Y302>C	A>G	RCV001040961, RCV001311380, RCV003147577	Pathogenic
	γ1	Y329 none reported			
	γ2	Y331>C	A>G	RCV000998485, RCV001858879	Pathogenic
	γ2	Y331>N	T>A	RCV002943951	Uncertain significance
	γ3	Y312>C	A>G	no report (rs1891306645)	Probably damaging (predicted)
	δ	A306 none reported			
	ε	Y336 none reported			
	π	C301>R	T>C	no report (rs1471896995)	Probably damaging (predicted)
	π	C301>S	G>C	no report (rs756455222)	Probably damaging (predicted)
	θ	C324>Y	G>A	no report (rs1556820226)	Benign (predicted)
	ρ1	Y340>C	A>G	no report (rs754011759)	Probably damaging (predicted)
	ρ2	Y320 none reported			
	ρ3	Y326>H	T>C	no report (rs895150804)	Probably damaging (predicted)
Glycine	α1	Y307>C	A>G	RCV000017441, RCV001376583, RCV005620337	Pathogenic
	α1	Y307>S	A>C	RCV000031892	Pathogenic
	α2	Y312 none reported			
	α3	Y312>D	T>G	no report (rs560093755)	Probably damaging (predicted)
	α3	Y312>H	T>C	no report (rs560093755)	Probably damaging (predicted)
	β	Y325>C	A>G	RCV003091751	Uncertain significance

bioRxiv preprint doi: https://doi.org/10.64898/2026.05.22.727206; this version posted May 26, 2026. The copyright holder for this preprint (which was not certified by peer review) is the author/funder, who has granted bioRxiv a license to display the preprint in perpetuity. It is made available under a CC-BY 4.0 International license.

Supplementary Table 3. Parameters for Hill equation fits to GABA concentration-response data

Receptor	EC_{50} (μM)	n_H	I_{max}	n	Figure
**GABA$_A$R**					
α1β2γ2	26 (21 to 32)	1.09 ± 0.09	0.828 ± 0.014	5	Fig. 2b
α1β2(Tyr277Cys)γ2	159 (116 to 227)	0.89 ± 0.09	0.147 ± 0.004	7	
α1β2γ2	95 (71 to 133)	0.92 ± 0.08	0.910 ± 0.024	6	Fig. 2c
α1(Tyr281Phe)β2γ2	42 (31 to 58)	0.93 ± 0.09	0.935 ± 0.027	10	
α1β2(Tyr277Phe)γ2	816 (471 to 1,740)	0.67 ± 0.08	0.334 ± 0.015	5	
α1β2γ2(Tyr292Phe)	57 (46 to 73)	1.01 ± 0.07	0.829 ± 0.019	5	
α1β2γ2	77 (63 to 95)	1.02 ± 0.07	0.773 ± 0.015	6	Fig. 3e
α1β2(Leu140Leu)γ2	62 (49 to 81)	1.48 ± 0.14	0.704 ± 0.015	6	
α1β2(Leu140Lah)γ2	322 (208 to 517)	0.84 ± 0.13	0.252 ± 0.008	7	
α1β3γ2	11 (10 to 12)	1.28 ± 0.05	0.830 ± 0.008	5	Suppl. Fig. 1b
α1β3(Tyr277Cys)γ2	82 (45 to 154)	1.23 ± 0.31	0.396 ± 0.023	6	
α1β3(Tyr277Phe)γ2	61 (53 to 70)	1.12 ± 0.06	0.509 ± 0.007	4	
α1β3γ2	102 (72 to 153)	0.84 ± 0.08	0.973 ± 0.030	3	Suppl. Fig. 1d
α1β3(Leu140Leu)γ2	76 (71 to 82)	1.20 ± 0.03	0.757 ± 0.006	6	
α1β3(Leu140Lah)γ2	261 (203 to 344)	0.93 ± 0.07	0.386 ± 0.008	8	
**GlyR**					
α1	186 (155 to 224)	1.70 ± 0.14	0.967 ± 0.020	6	Fig. 5b
α1(Tyr279Phe)	2,970 (1,790 to 6,980)	1.18 ± 0.22	0.446 ± 0.023	6	

Hill equation (Eq. 2) parameters are from fits to the normalized responses of the corresponding EC_{100} agonist plus propofol. EC_{50} is expressed as mean (95% coefficient interval), n_H and I_{max} are expressed as mean ± SEM, and n is the number of oocytes. There are multiple entries for wild type receptors, because they were run in parallel with the different mutants.

bioRxiv preprint doi: https://doi.org/10.64898/2026.05.22.727206; this version posted May 26, 2026. The copyright holder for this preprint (which was not certified by peer review) is the author/funder, who has granted bioRxiv a license to display the preprint in perpetuity. It is made available under a CC-BY 4.0 International license.

Supplementary Table 4. Organisms used for the phylogenetic analysis and number of corresponding protein sequences encoding pLGICs.

Organism	Code	Total sequences	Total Anionic	Anionic with Tyr	Total Cationic	Cationic with Tyr
Amphimedon queenslandica		**0**				
Mnemiopsis leidyi		**0**				
Homo sapiens	HUMAN	**43**	**23**	20	**20**	0
Drosophila melanogaster	DROME	**38**	**20**	13	**18**	2
Caenorhabditis elegans	CAEEL	**83**	**40**	37	**43**	2
Nematostella vectensis	NEMVE	**82**	**31**	31	**51**	10
Hydra vulgaris	HYDVU	**30**	**14**	11	**16**	0
Trichoplax adhaerens		**0**				
Salpingoeca rosetta		**0**				
Monosiga brevicollis	MONBE	**1**	**0**	0	**1**	0
Micromonas pusilla	MICPS	**2**	**1**	0	**1**	0
Micromonas commoda	MICCC	**1**	**1**	0	**0**	0
Emiliania huxleyi	EMIHU	**15**	**3**	1	**12**	6
Prymnesium parvum	PRYPA	**8**	**3**	0	**5**	2
Fragilariopsis cylindrus	FRCYL	**3**	**0**	0	**0**	0
Pseudo-nitzschia multistriata	PNMUL	**4**	**0**	0	**4**	0
Pseudo-nitzschia australis	PNAUS	**1**	**0**	0	**0**	0
Cylindrotheca closterium	CYCLO	**1**	**0**	0	**0**	0
Seminavis robusta	9STRA	**9**	**1**	0	**8**	0
Ditylum brightwellii	DIBRG	**2**	**0**	0	**0**	0
Aureococcus anophagefferens	AURAN	**13**	**9**	9	**4**	1
Pelagomonas calceolata	PLCAL	**12**	**2**	1	**10**	3
Euplotes crassus	EUPCR	**2**	**2**	0	**0**	0
Stylonychia lemnae	STYLE	**2**	**1**	0	**1**	0
Symbiodinium microadriaticum	SYMMI	**10**	**3**	2	**7**	5
Symbiodinium necroappetens	9DINO	**6**	**1**	1	**5**	4

bioRxiv preprint doi: https://doi.org/10.64898/2026.05.22.727206; this version posted May 26, 2026. The copyright holder for this preprint (which was not certified by peer review) is the author/funder, who has granted bioRxiv a license to display the preprint in perpetuity. It is made available under a CC-BY 4.0 International license.

References

Haloi, N., Eriksson Lidbrink, S., Howard, R. J., & Lindahl, E. (2025). Adaptive sampling-based structural prediction reveals opening of a GABAA receptor through the αβ interface. Science Advances, 11(2), eadq3788. https://doi.org/10.1126/sciadv.adq3788

Kleywegt, G. J., & Jones, T. A. (1996). Phi/psi-chology: Ramachandran revisited. Structure, 4(12), 1395–1400. https://doi.org/10.1016/s0969-2126(96)00147-5
